# Diagnostic Performance of the FilmArray® Meningitis/Encephalitis Panel for the Detection of Central Nervous System Infections in a Tertiary Care University Hospital in Tangier, Morocco

**DOI:** 10.7759/cureus.110050

**Published:** 2026-06-01

**Authors:** Nouhaila Chahid, Salma Lazraq, Reda Amrani Souhli, Majda El-Hassouni, Kawtar El Harrak, El Ghali Tazi, Fadila Bousgheiri, Adil Najdi, Karima Rissoul

**Affiliations:** 1 Microbiology and Virology Laboratory, Mohammed VI University Hospital, Tangier, MAR; 2 Microbiology, Faculty of Medicine and Pharmacy of Tangier, Abdelmalek Essaadi University, Tangier, MAR; 3 Epidemiology Laboratory, Faculty of Medicine and Pharmacy of Tangier, Abdelmalek Essaadi University, Tangier, MAR

**Keywords:** central nervous system infections, cerebrospinal fluid, cns infections, diagnostic performance, encephalitis, filmarray me panel, meningitis, meningitis/encephalitis panel, molecular diagnosis, multiplex pcr

## Abstract

Background: Central nervous system (CNS) infections represent serious medical emergencies. The development of syndromic tests has improved clinical management. The FilmArray® Meningitis/Encephalitis (ME) panel (BioFire Diagnostics, Salt Lake City, Utah) is an automated multiplex polymerase chain reaction (PCR) assay capable of simultaneously detecting 14 CNS pathogens from small cerebrospinal fluid (CSF) volumes. This study aimed to assess the diagnostic performance of this panel compared with conventional microbiological methods.

Methods: A retrospective diagnostic accuracy study was conducted in the microbiology laboratory of Mohammed VI University Hospital in Tangier, Morocco, between June 2023 and May 2025. Conventional methods included Gram staining, bacterial and fungal cultures, and cryptococcal antigen testing. The comparative analysis was limited to bacterial and fungal pathogens, using culture as the reference standard.

Results: Among the 402 samples analyzed, at least one pathogen was detected in 60 cases (14.9%). Viral pathogens were the most common (51.7%), followed by bacterial pathogens (40.0%), whereas fungal detection was rare (1.7%). Four cases of bacterial-viral co-detection (6.7%) were observed. For the detection of bacterial and fungal pathogens included in the panel, the FilmArray® ME panel demonstrated a sensitivity of 100%, a specificity of 94.9%, a positive predictive value (PPV) of 31.0%, and a negative predictive value (NPV) of 100%. Agreement between the FilmArray® ME panel and conventional culture was moderate (κ = 0.455), with culture confirmation obtained in 9 of 29 FilmArray®-positive bacterial and fungal cases.

Conclusion: The FilmArray® ME panel represents a valuable rapid adjunctive diagnostic tool for CNS infections. However, interpretation of its results should be integrated with clinical and conventional laboratory findings, particularly given its limited pathogen spectrum, including the absence of *Mycobacterium tuberculosis* detection.

## Introduction

Central nervous system (CNS) infections constitute a neurological emergency due to their high mortality and the risk of severe, potentially irreversible neurological sequelae if not promptly managed [1]. Rapid identification of causative pathogens is essential for optimal clinical management and for minimizing unnecessary exposure to broad-spectrum antimicrobial agents [2,3].

Conventional diagnostic approaches, including Gram staining and cerebrospinal fluid (CSF) culture, remain widely used [3,4]. Nevertheless, these methods have significant limitations, including long turnaround times and reduced sensitivity, particularly in patients with prior antimicrobial exposure or who present with a low pathogen load [3].

The FilmArray® Meningitis/Encephalitis (ME) panel is an automated multiplex polymerase chain reaction (PCR) assay that enables rapid, simultaneous detection of 14 CNS pathogens from small volumes of CSF, with results available in approximately one hour [3].

Despite its advantages, several challenges have been reported. Discrepancies between multiplex PCR results and conventional microbiological findings may occur, as previously described [5]. In addition, interpreting positive viral results can be challenging, particularly for viruses capable of latency or reactivation, such as human herpesvirus 6, for which the clinical relevance remains uncertain [6,7].

In North African settings, data on the use of multiplex molecular diagnostics for CNS infections remain limited. This study aimed to describe the distribution of pathogens detected by the FilmArray® ME panel and to evaluate its diagnostic performance for bacterial and fungal CNS infections compared with conventional microbiological methods.

## Materials and methods

Study design and setting

A retrospective diagnostic accuracy study was conducted at the microbiology laboratory of Mohammed VI University Hospital in Tangier, Morocco, between June 2023 and May 2025. During the study period, 2498 CSF specimens were received, of which 402 fulfilled the predefined inclusion criteria and were included in the final analysis.

Study population

Clinical suspicion of CNS infection was based on the presence of fever associated with neurological manifestations or, in neonates, nonspecific symptoms such as feeding difficulties or clinical deterioration [8]. Test ordering was performed according to routine clinical practice by the treating physicians.

Indications for FilmArray® ME panel testing included suspected meningitis with negative direct CSF examination, suspected herpetic encephalitis, CNS infection in immunocompromised patients, or compatible clinical presentations in pediatric patients.

Samples with insufficient CSF volume, duplicate specimens (only the first sample per patient was included), invalid FilmArray® results, or incomplete microbiological data were excluded from the study. Conventional microbiological culture results were available for all included CSF specimens.

Descriptive analysis included all pathogens detected by the FilmArray® ME panel. For comparative analysis, all 402 CSF specimens were considered. Diagnostic performance analysis was restricted to bacterial and fungal pathogens because no independent reference standard was available for viral targets. Viral-only detections identified by the FilmArray® ME panel were therefore classified as negative for bacterial/fungal targets in the comparative analysis.

Information regarding prior antimicrobial exposure before lumbar puncture was retrospectively collected from available medical records when documented. However, these data were incomplete for a proportion of patients.

Microbiological methods

CSF samples were transported promptly to the microbiology laboratory and processed according to standard laboratory protocols. Routine microbiological investigations included cytological examination, Gram staining, and bacterial and fungal cultures, which served as the reference standard for bacterial and fungal pathogen detection.

Specimens were inoculated onto blood agar and chocolate agar plates and incubated at 35-37 °C in a 5% CO₂ atmosphere for up to five days. Pathogen identification was performed using matrix-assisted laser desorption ionization-time-of-flight mass spectrometry (MALDI-TOF MS).

A cryptococcal antigen agglutination assay was performed when cryptococcal meningoencephalitis was clinically suspected.

FilmArray® ME panel

The FilmArray® ME panel (BioFire Diagnostics, Salt Lake City, Utah) was performed according to the manufacturer’s instructions and routine laboratory quality-control procedures. This automated syndromic multiplex PCR assay detects 14 bacterial, viral, and fungal pathogens directly from CSF samples and provides qualitative results within approximately one hour [3].

Statistical analysis

Demographic, clinical, and microbiological data were retrieved from the laboratory information system. Descriptive variables were expressed as frequencies, percentages, means, and standard deviations where appropriate.

Only bacterial and fungal results were included in the diagnostic performance analysis, using conventional culture as the reference standard. Sensitivity, specificity, positive predictive value, and negative predictive value were calculated. Agreement between the FilmArray® ME panel and culture results was assessed using Cohen’s κ coefficient and interpreted according to the Landis and Koch criteria [9]. Statistical analyses were performed using IBM SPSS Statistics for Windows, Version 27 (Released 2020; IBM Corp., Armonk, New York).

Ethical considerations

This study was conducted in accordance with the ethical principles of the Declaration of Helsinki and was approved by the Ethics Committee of Tangier University Hospital (Comité d’Éthique Hospitalo-Universitaire de Tanger; approval no. 252/26) on April 21, 2026.

## Results

Study population

Over the study period, 2498 CSF specimens were collected, of which 402 met the inclusion criteria and were included in the analysis. The study population comprised 218 males (54.2%) and 184 females (45.8%), with a male-to-female ratio of 1.18. The mean age was 14.2 years (SD, 21.0). Pediatric patients (<18 years) represented the majority of the cohort (73.4%, 295/402), whereas adults accounted for 26.6% (107/402).

Among the included patients, CSF samples most frequently originated from the emergency department (37.1%), followed by the intensive care unit (27.1%) and the pediatric department (25.4%). The most common clinical suspicion was meningitis (46.5%), followed by encephalitis (42.0%) and meningoencephalitis (11.4%). Data regarding antimicrobial exposure prior to lumbar puncture were incomplete and therefore analyzed only in available subgroups.

**Figure 1:**
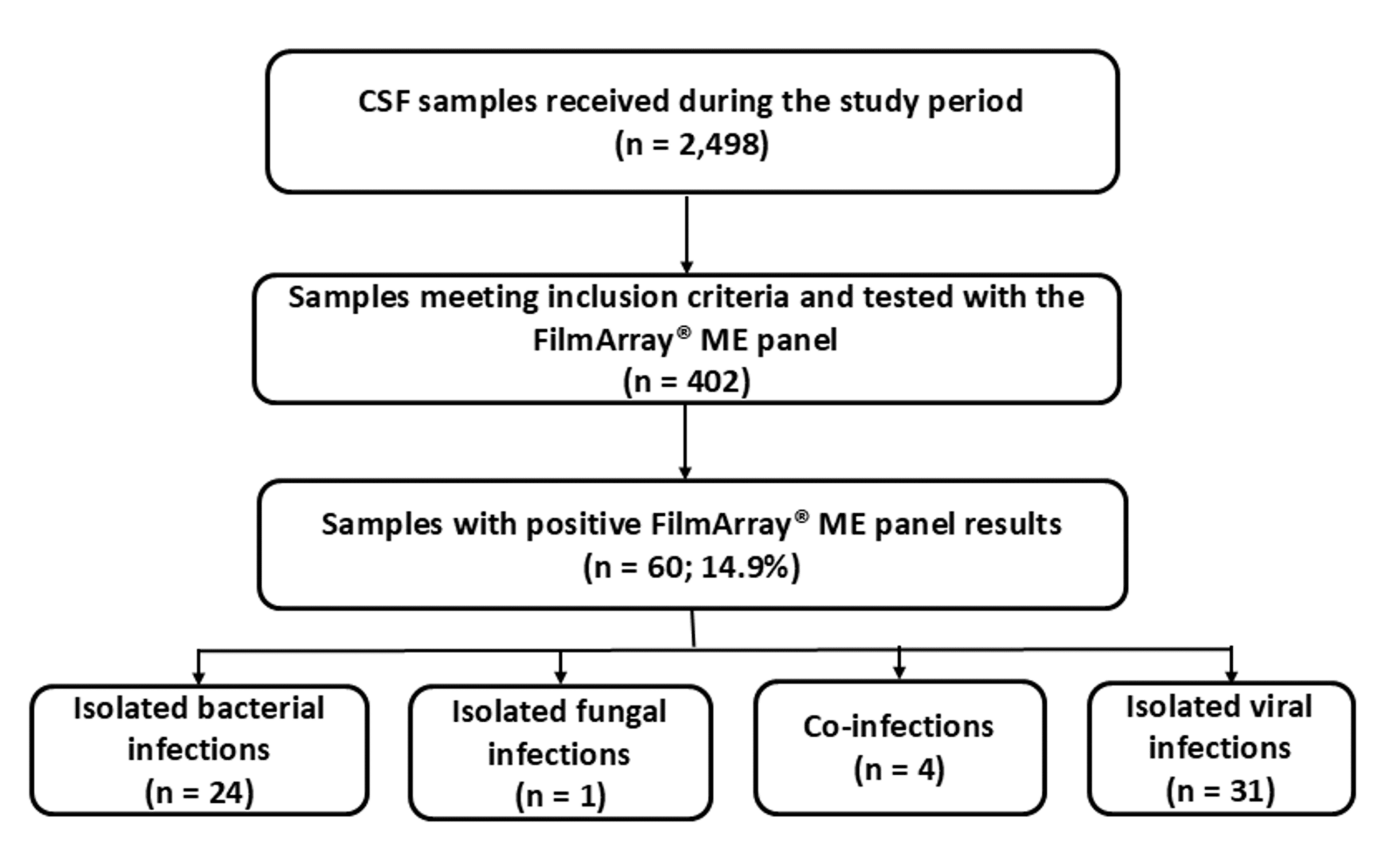
Flowchart illustrating the CSF sample selection and testing during the study period Of the 2498 cerebrospinal fluid (CSF) specimens received during this period, 402 fulfilled the predefined inclusion criteria and were subsequently analyzed using the FilmArray® ME panel. Among these, 60 (14.9%) samples tested positive, including isolated bacterial infections (n = 24), viral infections (n = 31), fungal infections (n = 1), and co-infections (n = 4).

The baseline demographic and clinical characteristics of the study population are summarized in Table 1, and the patient inclusion and selection process is illustrated in Figure 1. 

**Table 1:** Patient demographics and clinical characteristics (n = 402)

Variable	n (%)
Sex	
Male	218 (54.2)
Female	184 (45.8)
Age group	
Adult	107 (26.6)
Pediatric	295 (73.4)
Source of samples	
Emergency Department	149 (37.1)
Pediatric Department	102 (25.4)
Intensive Care Unit	109 (27.1)
Infectious Diseases Department	20 (5.0)
Neurology Department	13 (3.2)
Hematology Department	2 (0.5)
Neurosurgery Department	4 (1.0)
Cardiology Department	2 (0.5)
Ophthalmology Department	1 (0.2)
Clinical suspicion	
Meningitis	187 (46.5)
Encephalitis	169 (42.0)
Meningoencephalitis	46 (11.4)

Distribution of pathogens detected in CSF using the FilmArray® ME panel

Among the 402 CSF samples analyzed, 60 (14.9%) tested positive using the FilmArray® ME panel. Of these, 31 samples (51.7%) yielded viral pathogens only, 24 (40.0%) yielded bacterial pathogens only, and 1 sample (1.7%) yielded a fungal pathogen only. Four samples (6.7%) showed bacterial-viral co-detections. The detailed distribution of pathogens according to age groups is presented in Table 2.

**Table 2:** Age-stratified distribution of pathogens detected by the FilmArray® ME panel in positive CSF samples (n = 60) Counts exclude bacterial–viral co-detections, which are presented separately. HHV-6, human herpesvirus 6; EV, enterovirus; VZV, varicella-zoster virus; HSV-1, herpes simplex virus 1 ; HSV-2, herpes simplex virus 2 ; CMV, cytomegalovirus.

Pathogen	Total (n)	<2 months	2-23 months	2-15 years	16-35 years	36-65 years	>65 years
Bacteria							
*Streptococcus pneumoniae*	10	0	2	3	0	4	1
*Haemophilus influenzae*	9	2	4	1	2	0	0
* Neisseria meningitidis*	3	0	1	2	0	0	0
*Escherichia coli *K1	2	0	1	0	1	0	0
Viruses							
HHV-6	12	4	6	1	1	0	0
EV	6	1	2	2	1	0	0
VZV	5	1	0	3	0	1	0
HSV-1	4	0	2	2	0	0	0
CMV	3	1	0	0	1	1	0
HSV-2	1	0	0	0	0	1	0
Fungus							
*Cryptococcus neoformans*	1	0	0	0	1	0	0
Co-detections							
*S. pneumoniae* + CMV	1	0	0	0	0	1	0
*N. meningitidis* + HHV-6	1	0	1	0	0	0	0
*H. influenzae* + HSV-1	1	0	0	1	0	0	0
*H. influenzae* + HHV-6	1	0	0	1	0	0	0

Distribution of bacterial and fungal pathogens detected by the FilmArray® ME panel

Of the 29 CSF specimens positive for bacterial or fungal pathogens, CSF cytology showed pleocytosis in 22 cases (75.9%). Gram staining was positive in 3 samples (10.3%), including 2 cases of *S. pneumoniae* and 1 case of *H. influenzae*. Conventional culture was positive in 9 cases (31.0%), including 7 cases of *S. pneumoniae*, 1 *H. influenzae*, and 1 *Cryptococcus neoformans* isolate. Cryptococcal antigen testing in CSF was positive in the single case of *C. neoformans *infection (1/1, 100%). Analyses included both mono-detections and bacterial-viral co-detections involving bacterial or fungal pathogens.

Among the 402 CSF samples analyzed, an additional case of *Mycobacterium tuberculosis*, which is not included in the FilmArray® ME panel, was identified using GeneXpert MTB/RIF, despite a negative FilmArray result.

The distribution of bacterial and fungal pathogens detected by the FilmArray® ME panel, along with CSF cytology, Gram stain, culture results, and cryptococcal antigen findings, is summarized in Table 3. Detailed clinical, cytological, and microbiological characteristics of CSF specimens positive for bacterial and fungal pathogens are provided in the Appendices.

**Table 3:** Bacterial and fungal pathogens detected by the FilmArray® ME panel and corresponding CSF cytology, Gram stain, and culture results Counts include bacterial pathogens identified as mono- or co-detections (n = 4). Pleocytosis was defined according to age-adjusted cerebrospinal fluid white blood cell reference values [10,11]. NA, not applicable.

Microorganism	Positive FilmArray® ME panel (n)	CSF pleocytosis n (%)	Positive Gram stain n (%)	Positive culture n (%)
S. pneumoniae	11	11 (100)	2 (18.2)	7 (63.6)
N. meningitidis	4	4 (100)	0 (0)	0 (0)
H. influenzae	11	6 (54.5)	1 (9.1)	1 (9.1)
*E. coli* K1	2	0 (0)	0 (0)	0 (0)
C. neoformans	1	1 (100)	NA	1 (100)
Total	29	22 (75.9)	3 (10.3)	9 (31.0)

Comparison with conventional microbiological methods

All 402 CSF specimens included in the study had corresponding conventional culture results available. Among these, 29 specimens were positive for bacterial or fungal pathogens by the FilmArray® ME panel, including four bacterial-viral co-detections. These cases were included in the detailed concordance analysis between the FilmArray® ME panel and conventional culture.

Nine cases were culture-positive and considered concordant, whereas 20 cases were positive only by the molecular assay. No culture-positive/FilmArray-negative cases were identified. Overall, 20/29 (69%) bacterial/fungal FilmArray-positive cases were not confirmed by culture.

Most discordant cases involved *Haemophilus influenzae* (10/20, 50%), followed by *Neisseria meningitidis *and *Streptococcus pneumoniae *(each 4/20, 20%), and *Escherichia coli *K1 (2/20, 10%). Among the 20 discordant cases, three patients (15%) had received antimicrobial therapy prior to lumbar puncture. A detailed comparison between the FilmArray® ME panel and conventional culture is presented in Table 4.

**Table 4:** Comparison of the FilmArray® ME panel with conventional culture for bacterial and fungal pathogens Counts include bacterial pathogens identified as mono- or co-detections (n = 4). C+/F+, culture-positive/FilmArray-positive; C+/F−, culture-positive/FilmArray-negative; C−/F+, culture-negative/FilmArray-positive.

Pathogen	FilmArray-positive (n)	C+/F+	C+/F−	C−/F+
S. pneumoniae	11	7	0	4
N. meningitidis	4	0	0	4
H. influenzae	11	1	0	10
*E. coli* K1	2	0	0	2
C. neoformans	1	1	0	0
Total	29	9	0	20

Diagnostic performance of the FilmArray® ME panel

Using conventional culture as the reference standard, the FilmArray® ME panel demonstrated a sensitivity of 100%, a specificity of 94.9%, a positive predictive value (PPV) of 31.0%, and a negative predictive value (NPV) of 100% for the detection of bacterial and fungal pathogens only. Agreement with culture was moderate (κ = 0.455). Diagnostic performance data are summarized in Table 5.

**Table 5:** Diagnostic performance of the FilmArray® ME panel for bacterial and fungal pathogen detection compared with conventional culture Viral-only detections identified by the FilmArray® ME panel were classified as negative for bacterial/fungal targets in this comparative analysis, as conventional culture was not considered an appropriate reference standard for viral pathogens.

Variables	Culture-positive (n)	Culture-negative (n)	Total (n)
FilmArray® bacterial/fungal-positive	9	20	29
FilmArray® bacterial/fungal-negative	0	373	373
Total	9	393	402

## Discussion

In this retrospective analysis conducted at a university hospital in northern Morocco, at least one pathogen was detected in 14.9% of CSF samples using the FilmArray® ME panel. This positivity rate is slightly higher than that reported in a large multicenter study (8.7%) [3] and in a routine clinical setting (12.6%) [12]. These differences may be attributed to variations in patient populations, clinical indications for testing, and study design.

In our cohort, viral pathogens were the most frequently detected microorganisms, consistent with evidence that viruses constitute the most common etiological agents of CNS infections, particularly in pediatric and young adult populations [13,14]. Consistent with our data, viral pathogens were predominantly detected in pediatric patients, particularly those under 15 years of age. Rapid identification of a viral etiology, especially enteroviruses, has important clinical implications, including reducing unnecessary hospitalization, optimizing patient management, and improving healthcare resource utilization [15,16], as well as supporting antimicrobial stewardship by limiting inappropriate empirical antibiotic use and enabling timely therapeutic decisions [17].

In our cohort, human herpesvirus 6 (HHV-6) was the most commonly detected viral pathogen; however, some positive results may reflect viral reactivation or chromosomal integration rather than true neuroinvasive infection [18,19]. In addition, no quantitative HHV-6 PCR or plasma testing was available to distinguish active CNS infection from chromosomally integrated HHV-6 or incidental viral reactivation. Accordingly, the clinical relevance of HHV-6 detection in CSF remains controversial, highlighting the need to interpret molecular findings within the appropriate clinical context and pretest probability to avoid overdiagnosis [20,21].

*Streptococcus pneumoniae* and *Haemophilus influenzae *remain among the main bacterial pathogens identified, consistent with regional and international epidemiological data [22,23]. Bacterial-viral co-detections were identified in our cohort, a finding increasingly reported with multiplex molecular panels [3,21]. Their clinical significance remains uncertain and requires careful interpretation in light of clinical and laboratory findings. In our study, most co-detections were associated with features suggestive of bacterial meningitis, highlighting the importance of integrated interpretation of molecular and conventional laboratory findings.

The FilmArray® ME panel identified a case of *Cryptococcus neoformans* in an HIV-positive patient, consistent with cryptococcal antigen testing and fungal culture. Similar concordant findings have been reported in previous studies, although discrepancies with fungal culture have occasionally been observed [3].

Cytological analysis of CSF remains crucial in the diagnostic assessment of CNS infections. Although bacterial meningitis is typically characterized by neutrophilic pleocytosis, CSF findings may be normal, particularly early in the disease course [24,25]. In our cohort, most bacterial cases showed pleocytosis, while a substantial proportion exhibited normal or low cellularity, sometimes with lymphocytic predominance. Some of these atypical profiles were associated with bacterial-viral co-detections or prior antibiotic therapy. These findings indicate that normal or atypical CSF cytology does not exclude bacterial meningitis and highlight the complementary value of multiplex PCR for accurate pathogen detection.

Some bacterial FilmArray-positive cases, particularly those with acellular CSF findings, should be interpreted cautiously because true bacterial meningitis with normal CSF cellularity is uncommon. In the absence of systematic clinical adjudication, repeat lumbar puncture, or additional confirmatory molecular testing, false-positive molecular detections cannot be excluded in these discordant cases. This limitation is inherent to retrospective diagnostic accuracy studies using culture as an imperfect reference standard. 

Gram staining is a rapid and widely available technique for suspected bacterial meningitis, but its sensitivity depends on factors such as bacterial load and prior antibiotic exposure [26,27]. In our study, Gram staining was positive in only three CSF samples with confirmed bacterial infection, including two samples showing Gram-positive cocci that were subsequently identified as *Streptococcus pneumoniae* by culture.

CSF culture remains the reference standard for the diagnosis of bacterial meningitis, as it confirms infection, identifies the etiologic agent, and provides antimicrobial susceptibility data [10]. However, negative results may occur due to delayed sample transport affecting organism viability, low pathogen concentrations, or prior antimicrobial exposure [1,3].

In our study, among 29 samples with an identified bacterial or fungal pathogen, only 9 (31%) had positive CSF cultures. Among the 20 discordant FilmArray-positive/culture-negative cases, three patients had documented antimicrobial exposure prior to lumbar puncture. Although prior antimicrobial exposure explained some discordant cases, the high number of culture-negative/PCR-positive samples in untreated patients suggests that additional factors such as low pathogen burden or organism fragility also contributed to reduced culture sensitivity. Twenty cases (68.9%) were positive exclusively by the FilmArray® ME panel, a proportion higher than the 41% reported in a multicenter study [3], which may reflect differences in study design and testing indications. These observations further emphasize the added value of molecular diagnostics in routine clinical practice. This may be particularly relevant in resource-limited settings, where delayed diagnosis and empirical antimicrobial use remain major clinical challenges.

The FilmArray® ME panel showed good diagnostic performance in our cohort and appeared particularly useful for ruling out bacterial and fungal CNS infections. However, the relatively low positive predictive value should be interpreted cautiously, given the imperfect sensitivity of conventional culture and the higher analytical sensitivity of multiplex molecular assays. In addition, culture, as an imperfect reference standard, may yield false-negative results and lead to an underestimation of the true diagnostic performance of molecular assays [28,29].

These findings are in agreement with previous studies demonstrating improved pathogen detection using molecular methods, particularly in culture-negative cases and among patients who had received antibiotics prior to lumbar puncture [30, 31, 32]. Additionally, the implementation of multiplex PCR has been associated with shorter hospital stays and may contribute to more judicious antibiotic use through earlier etiologic diagnosis [33].

Several limitations should be noted. The FilmArray® ME panel targets only predefined pathogens, which may lead to false-negative results, particularly for *Mycobacterium tuberculosis*. In our cohort, one case of tuberculous meningitis, confirmed by the GeneXpert MTB/RIF assay, highlighted this limitation and emphasized the need for complementary diagnostic approaches. Additionally, HHV-6 DNA detection may not necessarily indicate active infection [18].

The relatively small number of positive cases (n = 60) may have limited the statistical power of subgroup analyses, particularly for less common pathogens. Accordingly, some subgroup findings should be interpreted cautiously. Additionally, the absence of a reference standard for viral detection limits the assessment of diagnostic performance. Therefore, molecular results should always be interpreted in conjunction with clinical presentation, CSF cytology, and conventional microbiological findings.

## Conclusions

The FilmArray® ME panel represents a valuable rapid adjunctive tool for the diagnosis of CNS infections, particularly in culture-negative cases. However, its results should be interpreted cautiously because conventional culture has limited sensitivity and the panel does not detect all potential pathogens, including *Mycobacterium tuberculosis*. Molecular findings should therefore be interpreted in conjunction with clinical presentation, CSF cytology, and conventional microbiological results.

Despite the relatively small sample size, this study provides important data from the North African context, where evidence regarding multiplex molecular diagnostics for CNS infections remains limited. Our findings highlight epidemiological and diagnostic trends broadly consistent with those reported in larger multicenter studies, while also demonstrating higher positivity and culture-discordance rates in our cohort.

## Appendices

**Table 6:** Clinical, cytological, and microbiological characteristics of cerebrospinal fluid samples positive for bacterial and fungal pathogens detected by the FilmArray® Meningitis/Encephalitis (ME) panel (n = 29). Bacterial–viral co-detections were identified in patients 11, 15, 25, and 26, corresponding to *S. pneumoniae *with cytomegalovirus (CMV), *N. meningitidis *with human herpesvirus 6 (HHV-6), *H. influenzae *with herpes simplex virus 1 (HSV-1), and *H. influenzae *with HHV-6, respectively. LP, lumbar puncture; CSF, cerebrospinal fluid; WBC, white blood cells; Neut, neutrophils; Lymph, lymphocytes; M, male; F, female.

Patient	Clinical diagnosis	Antibiotics before LP	Age/Sex	CSF WBC (cells/mm³); Neut (%); Lymph (%)	Gram stain	CSF culture	FilmArray® ME result
1	Meningitis	No	26 months/M	27; Neut 98%; Lymph 2%	Gram-positive diplococci	S. pneumoniae	S. pneumoniae
2	Meningitis	No	3 months/M	290; Neut 80%; Lymph 20%	Gram-positive diplococci	S. pneumoniae	S. pneumoniae
3	Meningitis	No	54 years/F	480; Neut 80%; Lymph 20%	Negative	S. pneumoniae	S. pneumoniae
4	Meningitis	No	5 months/M	32; Neut 62%; Lymph 38%	Negative	S. pneumoniae	S. pneumoniae
5	Meningitis	No	63 years/M	1184; Neut 95%; Lymph 5%	Negative	S. pneumoniae	S. pneumoniae
6	Meningitis	No	14 years/M	630; Neut 87%; Lymph 13%	Negative	Negative	S. pneumoniae
7	Meningitis	No	75 years/M	241; Neut 95%; Lymph 5%	Negative	S. pneumoniae	S. pneumoniae
8	Meningitis	No	57 years/F	1280; Neut 90%; Lymph 10%	Negative	Negative	S. pneumoniae
9	Meningitis	No	11 years/M	310; Neut 53%; Lymph 47%	Negative	Negative	S. pneumoniae
10	Meningitis	No	51 years/F	130; Neut 81%; Lymph 19%	Negative	Negative	S. pneumoniae
11	Encephalitis	No	57 years/F	720; Neut 55%; Lymph 45%	Negative	S. pneumoniae	S. pneumoniae
12	Meningitis with purpura fulminans	Yes	5 years/M	5120; Neut 81%; Lymph 19%	Negative	Negative	N. meningitidis
13	Meningitis	No	2 years/F	640 ; Neut 65% ; Lymph 35%	Negative	Negative	N. meningitidis
14	Febrile purpura	Yes	1 year/F	800 ; Neut 70% ; Lymph 30%	Negative	Negative	N. meningitidis
15	Meningitis	No	4 months/M	480 ; Neut 58% ; Lymph 42%	Negative	Negative	N. meningitidis
16	Meningitis	No	6 months/F	7360; Neut 80%; Lymph 20%	Gram-negative coccobacilli	H. influenzae	H. influenzae
17	Meningitis	No	26 years/M	14; Neut 70%; Lymph 30%	Negative	Negative	H. influenzae
18	Meningitis	No	4 years/M	895; Neut 30%; Lymph 70%	Negative	Negative	H. influenzae
19	Meningitis	No	1 day/F	1	Negative	Negative	H. influenzae
20	Meningitis	No	20 months/M	0	Negative	Negative	H. influenzae
21	Meningitis	No	20 months/M	0	Negative	Negative	H. influenzae
22	Meningitis	No	2 months/M	0	Negative	Negative	H. influenzae
23	Meningitis	No	2 years/M	7	Negative	Negative	H. influenzae
24	Meningitis	No	26 years/M	14; Neut 20%; Lymph 80%	Negative	Negative	H. influenzae
25	Meningitis	Yes	2 years/M	1	Negative	Negative	H. influenzae
26	Meningitis	No	7 years/M	5	Negative	Negative	H. influenzae
27	Meningitis	No	4 months/M	9	Negative	Negative	*E. coli* K1
28	Meningitis	No	55 years/M	1	Negative	Negative	*E. coli* K1
29	Meningoencephalitis	No	30 years/F	9	Negative	C. neoformans	C. neoformans
